# IGFBP-1 and IGFBP-2 are associated with a decreased pulse-wave velocity in young, healthy adults

**DOI:** 10.1186/s12872-021-01914-w

**Published:** 2021-03-11

**Authors:** Paul Pettersson-Pablo, Torbjörn K. Nilsson, Lars H. Breimer, Anita Hurtig-Wennlöf

**Affiliations:** 1 Department of Laboratory Medicine, Clinical Chemistry, Faculty of Medicine and Health, Örebro University Hospital, Södra Grevrosengatan 1, 703 62 Örebro, Sweden; 2 School of Medicine, Faculty of Medicine and Health, Örebro University, Örebro, Sweden; 3 Department of Medical Biosciences/Clinical Chemistry, Umeå University, Umeå, Sweden; 4 School of Health, Faculty of Medicine and Health, Örebro University, Örebro, Sweden; 5 The Biomedical platform, Department of Natural Science and Biomedicine, School of Health and Welfare, Jönköping University, Jönköping, Sweden

**Keywords:** Proteomics, Principal component analysis, cIMT, Vascular stiffness

## Abstract

**Background and aims:**

In healthy, young adults we analyzed a panel of cardiovascular disease related proteins in plasma and compared them with the vascular health of the subjects. The aim was to identify proteins with a relationship to the early atherosclerotic process in healthy individuals.

**Methods:**

We employed the proximity extension assay from OLINK proteomics to analyze 92 cardiovascular disease (CVD) related proteins on 833 subjects (men and women, ages 18–26). The women were further divided into an estrogen-using group and non-users. Protein expression was analyzed using principal component analysis (PCA). The following vascular examinations were performed: Pulse-wave velocity (PWV), augmentation index (AIX), carotid-intima media thickness (cIMT).

**Results:**

Three principal components were obtained using PCA to analyze the protein expression. None of the obtained principal components correlated significantly with AIX or cIMT. One of the components, explaining 6% of the total variance of the data, was significantly correlated with PWV. Upon examination of the proteins with the highest factor loadings on this component independently in a multivariable model, adjusting for established CVD risk biomarkers, insulin-like growth factor-binding protein 1 (IGFBP-1) and insulin-like growth factor-binding protein 2 (IGFBP-2) were found to independently, negatively correlate with PWV. Among the established risk factors included in the multivariable model, age was significantly and adversely correlated with all vascular measurements.

**Conclusions:**

In this population of healthy, young adults, groups of CVD related proteins correlate with PWV, but not AIX or cIMT. This group of proteins, of which IGFBP-1 and IGFBP-2 were independently, negatively correlated in a multivariable model with PWV, could have benificial effects on vascular stiffness. The robust association between age and PWV, AIX and cIMT provide insight into the impact of aging on the vasculature, which is detectable even in a population of young, healthy, non-smoking individuals of ages spanning only 8 years.

**Supplementary information:**

The online version contains supplementary material available at 10.1186/s12872-021-01914-w.

## Introduction

While cardiovascular disease (CVD) predominantly affects the elderly, the atherosclerotic progression begins in childhood [1]. Endothelial dysfunction occurs early in atherosclerosis and may precede manifest CVD by decades [2]. An increased pulse-wave velocity (PWV) in childhood, a measurement of vascular stiffness, is associated with an increased risk of later developing CVD [3]. Traditional risk factors such as dyslipidemia, increased blood pressure and obesity are commonly observed in a clinical setting involving adult CVD patients. These risk factors, set already in childhood may confer an increased risk of early CVD [4]. However, the first CVD events often affect individuals without any known risk factors who would be classified as having a low risk according to risk assessment tools based on traditional risk factors [5]. An improved understanding of atherosclerotic pathophysiology in its early stages could thus contribute towards a sharpening of the predictive accuracy of risk assessment in young individuals who develop CVD despite a lack of traditional risk factors [6].

Proteomics analyses, incorporating larger panels of proteins, have the potential to identify clinically important sets of proteins and overcome the limitations of studying individual proteins, which has often been found to offer limited predictive power [7, 8]. Previous studies on vascular stiffness and thickness variables that employ serum or plasma proteomics have found differences in the high-density lipoprotein (HDL) proteome between subjects with a high and low PWV, respectively [9] and differences in nitric oxide related metabolites related to differences in PWV and central systolic blood pressure among black and white subjects [10]. The subjects recruited in the Lifestyle, Biomarkers and Atherosclerosis study (LBA) are young and healthy. In this exploratory study, we measured PWV, augmentation index (AIX) and carotid intima-media thickness (cIMT) and compared them to the OLINK CVD III proteomics panel (Olink Proteomics, Uppsala, Sweden) that measures the plasma concentrations of 92 proteins associated with CVD. Our aim was to identify proteins or groups of proteins associated with the earliest stages of vascular dysfunction and remodeling. As estrogen containing contraceptives have been shown to influence plasma protein concentrations[11], a secondary aim was to examine the estrogen effect on protein expression by splitting female subjects into two groups based on whether they were using contraceptives.

## Subjects and methods

### Study population

The LBA study is a cross-sectional study on 833 Swedish young adults between 18 and 26 years of age. The recruitment process has been described previously [12]. Upon enrollment, subjects filled out a health form including on family history of CVD or diabetes. Subjects reporting chronic disease such as diabetes mellitus or Crohn’s disease were excluded. One individual reported having quit smoking 2 weeks prior to data collection was excluded. Questionnaire data regarding family history of CVD suffered from a high amount of missing values (22%) due to subjects being unable to respond, citing unfamiliarity with whether their family had any such history or not. Body fat percentage was measured using a bioelectrical impedance body composition analyzer (Tanita BC-418 MA; Tanita Europe B.V., Amsterdam, the Netherlands). Adjustments were made with 1 kg for clothes and the standard setting was used.

### Vascular examinations

Three physiological vascular examinations were used, PWV, AIX and cIMT, to examine the vascular function and structure of the subjects. The examination procedure has been previously described in detail [12, 13]. In brief, all measurements were repeated three times and the average was used in the statistical models. Repeatability measures (standard deviation) were: 0.44 for PWV, 2.15 for AIX and 0.017 for cIMT. PWV and AIX were measured using applanation tonometry with a SphygmoCor device (AtCor Medical Pty Ltd, SphygmoCor, Sydney, Australia) on subjects having rested for at least 20–30 min. For AIX, the radial artery tonometry was performed at the subject’s right wrist. AIX was calculated from the aortic pressure waveform and adjusted to a heart rate of 75 beats per minute. cIMT was measured using a high-resolution ultrasound B mode system, (GE Healthcare, Vivid E9, Chicago, Illinois, US) with a 12 MHz linear array transducer. The measurement was made 10 mm proximally of the carotid bulb with the structures identified by the semi-automated Vivid E9 edge detection software.

### Serum CVD risk markers in adjusted models

In our statistical analyses, we included adjustment for the following CVD biomarkers: age, sex, body fat percentage, Apolipoprotein B/Apolipoprotein A-1 ratio (Apo B/ApoA-1 ratio), Homeostatic Model Assessment for Insulin Resistance (HOMA-IR) and C-reactive protein (CRP) to examine the independency of any relationships found between proteomics analyses and vascular examinations. These adjustments were included for being well-established as biomarkers of increased CVD risk [14, 15]. It is known that a subset of the population display discordance between low-density lipoprotein (LDL) and Apo B concentrations [16] so we measured both Apo B and Apo A-1 LDL and HDL and to examine whether the choice of lipid biomarkers would lead to any differences in the associations found in the multivariable models. Fasting serum samples were collected using vacutainer tubes (BD Vacutainer; BD AB, Stockholm, Sweden) with a thrombin clot activator. Serum was left to clot for at least 30 min before centrifugation and subsequent analysis. Insulin was analyzed with the Abbott Architect Insulin Assay, a sandwich immunoassay using chemiluminescence detection with a coefficient of variation (CV) of 7% at 8 mIU/L on an Architect i2000SR unit (Abbott, Abbot Park, IL, USA). Insulin and glucose were used in the calculation of the HOMA-IR [17]. CRP and Apo A-1 and Apo B were analyzed on a Siemens ADVIA 1800 Chemistry instrument. The Apo A-1 assay had a CV of 4% at 0.9 g/L and the Apo B assay a CV of 5% at 1,5 g/L. CRP had a CV of 5% at 0.74 mg/L with the Siemens High Sensitivity CRP Assay. Direct LDL was assayed by a two-step colorimetric assay with Vitros MicroWell technology. HDL (6% CV at 1.0 mmol/L), LDL (5% CV at 2.4 mmol/L) and glucose (4% CV at 4.6 mmol/L) were analyzed on a Vitros 5.1 system (Vitros 5.1TM FS, Clinical Chemistry Instruments, Raritan, NJ, USA). Direct HDL, triglycerides (TG), and glucose were assayed colorimetrically with Vitros MicroSlide technology (5.1TM FS; Clinical Chemistry Instruments, Raritan, NJ, USA).

### Proteomic analysis by proximity extension assay (PEA)

Fasting plasma samples were collected into ethylenediaminetetraacetic acid (EDTA) plasma vacutainer tubes (BD Vacutainer; BD AB, Stockholm, Sweden). Analysis was performed using PEA technology (OLINK® Proteomics, Uppsala, Sweden), using antibodies marked with oligonucleotides to permit the simultaneous measurement of several analytes in a sample. Antibody pairs with complementary oligonucleotide strands bind to an analyte and cross-reactivity is kept to a minimum as amplification requires complementary antibodies to take place. The results of the measurement are presented as normalized protein expression (NPX). NPX is a normalized value reflecting the relative concentration of the proteins among the samples in the run. We employed the CVD III panel (www.olink.com) to measure the relative concentrations of 92 CVD related proteins [18].

### Statistical analyses

Statistical analyses were performed in SPSS ver 25 (IBM Corp., Armonk, NY, USA) and R ver 3.4.3 (R Foundation for Statistical Computing, Vienna, Austria). The risk factors used in the adjusted models were entered as continuous z score transformed normalized variables, except for age, sex and contraceptive use in women. Principal component analysis (PCA) was used to achieve manageable data reduction and identify components to replace proteins that were highly correlated. Scree plot visualization was used to assess the number of principal components (PC) to retain. We used varimax oblique rotation to minimize the number of variables with high factor loadings. In multivariable models, PCs were compared with vascular measurements in analyses adjusted for age, sex, body fat percentage, Apo B/Apo A-1 ratio, HOMA-IR and CRP and contraceptive use among women. The proteins with the highest factor loadings on the PCs were also examined separately in multiple linear regression models to examine their correlation with vascular measurements independently of the PCA. Because of the exploratory nature of the present study, we did not make statistical adjustments to account for multiple comparisons.

## Results

### Sample baseline characteristics

The clinical characteristics of the LBA population in shown in Table 1. The population comprises more women than men (576 vs. 257). In Table 1, statistically significant mean differences of traditional risk factors such as LDL and PWV and protein expression shown as PCs can be seen between men and women, the latter divided into estrogen and non-estrogen using women. Non-estrogen using women had a statistically significant higher expression of PC 1 and a lower expression of PC 3 than estrogen users.

**Table 1:** Baseline characteristics of the studied population sample

	Men (n = 257)	Non-estrogen using women (n = 427)	Estrogen using women (n = 149)	*p* value M vs NEU	*p* value M vs EU	*p* value NEU vs EU
Age	22 ± 2.0	22 ± 2.0	22 ± 1.6	0.69	0.27	0.58
Body fat (%)	15 ± 5.6	28 ± 6.8	27 ± 5.9	< 0.001	< 0.001	0.42
LDL (mmol/L)	2.3 ± 0.69	2.2 ± 0.69	2.5 ± 0.79	0.36	< 0.001	< 0.001
HDL (mmol/L)	1.2 ± 0.28	1.4 ± 0.35	1.5 ± 0.41	< 0.001	< 0.001	0.81
CHOL (mmol/L)	4.0 ± 0,79	4.3 ± 0.77	4.4 ± 0.77	0.0034	< 0.001	0.0042
Systolic BP (mmHg)	122 ± 11	109 ± 9.0	112 ± 7.5	< 0.001	< 0.001	0.0034
Diastolic BP (mmHg)	64 ± 6.7	64 ± 6.0	65 ± 6.9	0.089	0.75	0.015
TG (mmol/L)	0.79 ± 0.35	0.75 ± 0.32	0.99 ± 0.39	0.25	< 0.001	< 0.001
Fasting serum insulin (mIE/L)	7.5 ± 3.7	8.1 ± 4.7	8.2 ± 4.4	0.32	0.56	0.99
HOMA-IR	1.8 ± 0.90	1.8 ± 1.1	1.7 ± 0.93	0.95	0.99	0.96
Apo B (g/L)	0.77 ± 0.18	0.78 ± 0.18	0.83 ± 0.20	0.97	< 0.001	< 0.001
Apo A-1 (g/L)	1.4 ± 0.21	1.5 ± 0.27	1.7 ± 0.325	< 0.001	< 0.001	< 0.001
Apo B/Apo A-1 ratio	0.56 ± 0.14	0.51 ± 0.14	0.50 ± 0.15	< 0.001	0.032	0.75
CRP (mg/L)	1.3 ± 2.7	1.4 ± 2.8	4.1 ± 7.1	0.99	< 0.001	< 0.001
PWV (m/s)	5.6 ± 0.86	5.2 ± 0.69	5.3 ± 0.9	< 0.001	0.092	0.0045
AIX (%)	− 8.4 ± 9.5	− 5.0 ± 10	− 4.7 ± 9.1	< 0.001	0.0019	0.99
cIMT (mm)	0.60 ± 0.073	0.49 ± 0.057	0.50 ± 0.055	0.48	0.18	0.61
PC 1	0.64 ± 0.75	− 0.020 ± 0.87	− 1.0 ± 0.85	< 0.001	< 0.001	< 0.001
PC 2	0.032 ± 0.97	0.036 ± 1.1	− 0.16 ± 0.86	0.99	0.29	0.21
PC 3	− 0.52 ± 0.78	− 0.068 ± 0.86	1.1 ± 0.87	< 0.001	< 0.001	< 0.001

Values are presented as mean ± SD (standard deviation). P value: comparison between groups by ANOVA with Tukey post hoc comparison. M = Men. NEU = Non-estrogen using women. EU = estrogen using women. LDL = low-density lipoprotein. HDL = high-density lipoprotein. TG = triglycerides. CHOL = total cholesterol. BP = blood pressure. Apo B = apolipoprotein B. Apo A-1 = Apolipoprotein A-1. CRP = C-reactive protein. BP = Blood pressure. PWV = pulse-wave velocity. AIX = Augmentation index. cIMT = carotid-intima media thickness. PC = principal component

### Protein expression and PCA

The PCA was conducted with varimax orthogonal rotation. A scree plot showed inflexions justifying extraction of three PCs, altogether explaining 45% of the total variance of the 92 proteins in the panel (Table 2). The factor loadings of each individual protein on the three extracted PCs after rotation are shown in Additional file 1: Table 1. The PCs were entered in a multivariable linear regression model to examine their possible correlations with PWV, AIX and cIMT. Serum lipid biomarkers, glucose, insulin and CRP were included in the model to assess whether the correlations found were independent of the effect of the established biomarkers of CVD. Inclusion of HDL and LDL instead of apolipoprotein B/apolipoprotein A-1 ratio in the model to represent the serum lipids did not considerably impact the multivariable model (similar β coefficients and *p* values; not shown). As can be seen in Table 3, PC 3 was significantly correlated with PWV in the multivariable model. Inclusion of the family history of CVD variable in the multivariable model attenuated the strength of the association, decreasing the β coefficient of PC 3 (from 0.11 to 0.060), increasing the *p* value to non-significance (from 0.034 to 0.26). The 168 missing values from the questionnaire on heredity could have contributed to a loss of power. Neither AIX nor cIMT showed any significant associations with any of the PCs (Table 3). Age was significantly and adversely correlated with all vascular measurements.

**Table 2:** Squared loadings and total variance of the 92 proteins in the proteomics analysis that is explained by each of the three principal components extracted by the principal component analysis. Results shown after varimax oblique rotation

Component	Squared loadings	% of Variance	Cumulative %
1	24.25	26.36	26.36
2	11.78	12.81	39.17
3	5.56	6.05	45.23

**Table 3:** Multivariable linear regression: Vascular measurements as a function of principal components and established risk factors of cardiovascular disease

	PWV (R^2^: 0.071)		AIX (R^2^: 0.041)		cIMT (R^2^: 0.019)	
	β (95% CI)	*p*	β (95% CI)	*p*	β (95% CI)	*p*
PC 1	− 0.067 (− 0.15;0.020)	0.17	− 0.040 (− 0.13;0.052)	0.66	0.079 (− 0.10;0.043)	0.097
PC 2	0.013 (− 0.066;0.093)	0.76	0.032 (− 0.051;0.12)	0.48	− 0.040 (− 0.077;0.069)	0.35
PC 3	0.11 (0.027;0.20)	0.023*	0.065 (− 0.024;0.15)	0.42	− 0.013 (− 0.042;0.0.11)	0.71
Age	0.091 (0.051;0.13)	< 0.001*	0.075 (0.032;0.12)	< 0.001*	0.045 (0.011;0.080)	0.041*
Sex	0.10 (− 0.11;0.29)	0.38	0.098 (− 0.12;0.31)	0.57	− 0.041 (− 0.15;0.18)	0.67
Body fat%	0.10 (0.021;0.16)	0.022*	0.086 (− 0.0011;0.17)	0.13	− 0.0091 (− 0.014;0.13)	0.78
Apo B/Apo A1	0.044 (− 0.043;0.13)	0.33	− 0.00075 (− 0.091;0.093)	0.98	0.077 (− 0.052;0.088)	0.11
HOMA− IR	− 0.067 (− 0.11;0.022)	0.16	− 0.059 (− 0.16;0.041)	0.24	− 0.036 (− 0.079;0.056)	0.48
CRP	0.012 (− 0.81;0.11)	0.80	0.061 (− 0.038;0.16)	0.23	0.015 (− 0.087;0.17)	0.78

Variables are separated on sex and z-score transformed before inclusion in the multivariable model. PWV = Pulse-wave velocity. AIX = Augmentation index. cIMT = carotid intima media thickness. β = Beta coefficient. PC = Principal component. Apo B/ApoA-1 = Apolipoprotein B/Apolipoprotein A-1 ratio. TG = Triglycerides. HOMA-IR = Homeostatic model assessment insulin resistance. CRP = C-reactive protein. * indicates a *p* value < 0.05

**Table 4:** Multivariable linear regression. PWV as a function of the proteins with factor loadings (> 0.4) on principal component 3. Established risk factors of atherosclerosis are included in the multivariable model

	R^2^	β (95% CI)	*p* value
TFF3 + Model 1	0.063	0.036 (− 0.019; 0.90)	0.20
GDF-15 + Model 1	0.062	0.11 (− 0.062;− 0.029)	0.20
RARRES2 + Model 1	0.065	0.23 (0.0081;0.45)	0.058
IGFBP-2 + Model 1	0.069	− 0.18 (− 0.29;− 0.065)	0.0022*
CD166 + Model 1	0.062	0.18 (− 0.087;0.44)	0.19
IGFBP-1 + Model 1	0.068	− 0.10 (− 0.18;− 0.017)	0.018*
OPG + Model 1	0.061	0.091 (− 0.12;0.30)	0.39
MMP-3 + Model 1	0.060	− 0.0021 (− 0.12;0.12)	0.98
AZU1 + Model 1	0.061	− 0.029 (− 0.17;11)	0.68
ST2 + Model 1	0.062	− 0.014 (− 0.13;0.10)	0.81
CCL15 + Model 1		0.12 (− 0.0069;0.25)	0.062

Model 1 includes age, sex, body fat%, Apolipoprotein B/Apolipoprotein A-1 ratio, Homeostatic model assessment insulin resistance (HOMA-IR) and C-reactive protein (CRP). TFF3 = Trefoil factor 3. GDF-15 = Growth/differentiation factor 15. RARRES2 = Retinoic acid receptor responder protein 2. IGFBP-2 = Insulin-like growth factor-binding protein 2. CD166 = CD166 antigen. IGFBP-1 = Insulin-like growth factor-binding protein 1. OPG = Osteoprotegrin. MMP3 = Matrix metalloproteinase-3. AZU1 = Azurocidin. ST2 = ST2 protein. CCL15 = C–C motif chemokine 15. * indicates a p value < 0.05.

The proteins having factor loadings higher than 0.4 on PC3 were, in descending order, trefoil factor 3 (TFF3), growth/differentiation factor 15 (GDF-15), retinoic acid receptor responder protein 2 (RARRES2), insulin-like growth factor-binding protein 2 (IGFBP-2), CD166 antigen (CD166), insulin-like growth factor-binding protein 1 (IGFBP-1), osteoprotegrin (OPG), matrix metalloproteinase-3 (MMP3), azurocidin (AZU1), ST2 protein (ST2), and C–C motif chemokine 15 (CCL15) (Additional file 1: Table 1). To further explore these proteins and their possibly independent capacity to predict an increased PWV, we employed multivariable analyses in which these collinear proteins were separately examined for their correlations with PWV when controlling for established CVD risk factors. IGFBP-1 and IGFBP-2 were found to be significantly, inversely correlated with PWV (Table 4).

## Discussion

In this study we examined the relationship between the expression of CVD related proteins and vascular function and structure in healthy, young adults. PCA identified 3 clusters of collinear CVD related proteins. PC 3 showed a strong independent correlation with PWV (Table 3), despite explaining only 6% of the total variance of our proteomics data. Among the proteins with the highest factor loadings on PC 3, IGFBP-1 and IGFBP-2 were inversely correlated with PWV.

The insulin-like growth factors (IGFs) constitute a group of proteins with structural and functional similarity to insulin [19]. Their activity is modulated by IGFBPs, such as IGFBP-1 and IGFBP-2. Upon sequestering IGF ligands from their receptors the binding proteins inhibit IGF mediated signaling in a variety of tissues [20]. In addition to their IGF binding properties, they have been suggested to exert IGF-independent regulatory activity on extracellular matrix tissue [21]. Proteolytic counteraction of IGFBP activity provides another layer of regulation of IGF related pathways. Alterations in IGFBP proteolysis has been implicated in various diseases, such as cancer and inflammation [22, 23].

In this study, a higher protein expression of IGFBP-1 and IGFBP-2 in plasma was associated with a lower vascular stiffness, assessed as PWV. Studies on the function of the IGF binding proteins suggest that IGFBP-2 is the IGFBP which is primarily involved in regulatory activity in tissues throughout the body [20]. A study on 379 men between the ages of 30–65 who underwent a one year lifestyle intervention program observed an association between low baseline concentrations of IGFBP-2 and a disadvantageous metabolic status, in particular with respect to lipoprotein lipid profile [24]. This coincides with various studies finding a correlation between IGFBP-2 and serum lipids [25–27]. Conversely, in many studies a relationship between an increased IGFBP-2 and an increased CVD risk was found [28, 29]. In our cohort, the inclusion of lipoprotein biomarkers apo B and apo A-1, HDL and LDL cholesterol concentration and serum insulin concentration did not impact the robust correlation between IGFBP-1 and IGFBP-2 and PWV (Table 4), which suggests that the effects these proteins exert on vascular stiffness are not only due to their interplay with serum lipids or insulin but by other metabolic pathways.

The PCA established three clusters of proteins that together explained a fairly high percentage (45%) of the variance in our proteomics data. Several of the proteins highly influencing the characteristics of PC 3 (Table 4) have been implicated in CVD risk. TFF3 has been implicated in kidney injury and is speculated to be involved in cell repair in inflammation [30, 31]. Similarly, GDF-15 has shown an association with CVD. Depending on the study, an increased GDF-15 concentration has been suggested to be both beneficial and detrimental. Its CVD related effects have been hypothesized to be related to inflammation and tissue injury and repair [32, 33]. RARRES2 (chemerin) is mainly expressed in adipose tissue and has been implicated in obesity, glucose metabolism and inflammation [34]. In this study, upon examining the PC 3 proteins independently in multivariable regression models, only IGFBP-1 and IGFBP-2 showed independent, significant correlations with PWV. The inverse correlation suggests that subjects with high concentrations in plasma of these proteins have a favorable vascular function. The results may be indicative of the intricate but subtle interplay in the relationship between vascular phenotype and plasma proteins in a population composed of healthy, non-smoking individuals. Such a population would be thought to have only minor vascular changes (as seen by the low means and standard deviations of the vascular variables in Table 1) and to correlate only weakly with potential risk factors. Only the vascular stiffness measurement PWV showed a significant, inverse relationship with PC 3, while neither AIX nor cIMT was significantly correlated with any PCs. One study assessing CVD risk using the Framingham risk score found that it correlated better in young men with PWV and cIMT than with AIX [35]. Vascular smooth muscle cells differ in mechanical properties and protein expression based on their location in arterial tree [36], which could provide an explanation to the difference in protein expression found between the vascular measurements we used. While vascular function measurements are usually highly correlated, a lack of correlation between different methods with respect to a certain outcome has been reported. This is likely due to either to the differences in locality of the measures, differences in the physiological properties examined (i.e. blood flow vs wave reflection), or hardware or software related differences of the equipment and algorithms used for calculating the result [37]. PWV being the gold-standard of vascular stiffness measurements and possibly a more sensitive marker in the earliest stages of vascular pathology could explain the discrepancy we found in the relationship between proteome and vascular variables.

A consistent finding in this study was the pervasive association observed between age and all three studied markers of vascular stiffness and thickness, despite the narrow range of 18–26 years in the cohort. Most of the *R*^2^ of the multivariable models were owing to the age variable. The effect of aging on vasculature has been examined in more detail in recent studies, for instance a linear increase of cIMT over the course of a lifetime has been demonstrated, in all age spans and in both individuals with and without risk factors [38, 39]. PWV is similarly dependent on age [40]. Our results match these findings while underlining how even a few years measurably impact vascular status, even when adjusted for established risk biomarkers.

While the mean PWV, AIX and cIMT differed between groups of men, non-estrogen using women and estrogen using women in the population (Table 1), sex was not significantly associated with any of these vascular measurements in the multivariable models (Table 3). In this study, estrogen contraceptive users had a higher mean PWV (Table 1) and lower PC 1 and higher PC 3 when comparing means (Table 1). It is possible that the regulatory properties in inflammation and tissue repair of the proteins corresponding to these PCs could in part explain the increased vascular stiffness observed in other studies in contraceptive using women [41, 42].

The main limitation of this study is its cross-sectional design proscribing inferences of causality in the relationship between plasma protein expression and the physiological measurements. Due to the exploratory nature of this study, we made the choice not to make any adjustment for multiple testing to promote hypothesis generating findings that could be explored in further studies. The majority of the subjects were university students at Örebro University, so we were not able to examine or adjust for differences in socioecomonic position in this study. Among the strengths of the study is the fairly large population consisting of an under-studied population subgroup in the context of cardiovascular research: healthy, young, non-smoking individuals in various stages of vascular stiffness and thickness. The proteins that were found to correlate with vascular stiffness in this study need to be examined in further studies of longitudinal design to examine the possibly causal relationships between the proteins implicated here and a CVD risk.

## Conclusion

In young, healthy individuals, we employed PCA to identify groups of CVD related plasma proteins and examined their relationship with PWV. One of the PCs, whose properties were determined by proteins implicated in inflammation, metabolic regulation and tissue repair was independently associated with PWV. Among these proteins, IGFBP-1 and IGBFP-2 were independently associated with PWV suggesting that high concentrations of these proteins have beneficial properties with respect to vascular stiffness. The robust associations between age and increased PWV, AIX and cIMT provide insight into the impact of aging on the vasculature, which is detectable even in a population of young, healthy, non-smoking individuals of ages spanning only 8 years.

## Supplementary Information

**Additional file 1.** **Supplementary Table 1.**Component matrix showing the three principal components that were extracted from the PCA of the 92 protein proteomics and their makeup of the individual proteins listed according to their factor loadings scores (AKA component loadings). For ease of reading, factor loading scores below 0.3 have been censored.

## Data Availability

The datasets generated and/or analysed during the current study are not publicly available due to research subject confidentiality but are available in a de-identified form from the corresponding author on reasonable request and with permission of the PI of the study.

## Abbreviations

AIX: Augmentation index
Apo A-1: Apolipoprotein A-1
Apo B: Apolipoprotein B
AZU1: Azurocidin
BP: Blood pressure
CCL15: C–C motif chemokine 15
CHOL: Total cholesterol
cIMT: Carotid intima-media thickness
CRP: C-reactive protein
GDF-15: Growth/differentiation factor 15
HDL: High-density lipoprotein
IGFBP: Insulin-like growth factor binding protein
HDL: High-density lipoprotein
HOMA-IR: Homeostatic model assessment, insulin resistance
LDL: Low-density lipoprotein
MMP3: Matrix metalloproteinase-3
OPG: Osteoprotegrin
PC: Principal component
PCA: Principal component analysis
PWV: Pulse-wave velocity
RARRES2: Retinoic acid receptor responder protein 2
ST2: ST2 protein
TFF3: Trefoil factor 3
TG: Triglycerides
