# Supplementary material for: IGFBP-1 and IGFBP-2 are associated with a decreased pulse-wave velocity in young, healthy adults
Source: BMC Cardiovasc Disord. 2021 Mar 11;21:131. doi: 10.1186/s12872-021-01914-w (PMC7949246; doi:10.1186/s12872-021-01914-w)
Supplement: Supplementary file 1 — Additional file 1. Supplementary Table 1.Component matrix showing the three principal components that were extracted from the PCA of the 92 protein proteomics and their makeup of the individual proteins listed according to their factor loadings scores (AKA component loadings). For ease of reading, factor loading scores below 0.3 have been censored. [file 12872_2021_1914_MOESM1_ESM.docx]

**IGFBP-1 and IGFBP-2 are associated with a decreased pulse-wave velocity in young, healthy adults**

Paul Pettersson-Pablo^abc*^, Torbjörn K Nilsson^c^, Lars H Breimer^ab^, Anita Hurtig-Wennlöf^d^.

*^a^Department of Laboratory Medicine, Faculty of Medicine and Health, Örebro University Hospital, Örebro, Sweden.*

*^b^School of Medicine, Faculty of Medicine and Health, Örebro University, Örebro, Sweden.*

*^c^Department of Medical Biosciences/Clinical Chemistry, Umeå University, Umeå, Sweden.*

*^d^School of Health, Faculty of Medicine and Health, Örebro University, Örebro, Sweden.*

*Corresponding author. Email: paul.pettersson-pablo@regionorebrolan.se
Postal address: Paul Pettersson-Pablo, Department of Laboratory Medicine, Clinical chemistry, Örebro University Hospital, Södra Grevrosengatan 1, 703 62 Örebro, SWEDEN.

paul.pettersson-pablo@regionorebrolan.se

torbjorn.nilsson@umu.se

lars.breimer@regionorebrolan.se

anita.hurtig-wennlof@oru.se

**Supplementary Table 1**

Component matrix showing the three principal components that were extracted from the PCA of the 92 protein proteomics and their makeup of the individual proteins listed according to their factor loadings scores (AKA component loadings). For ease of reading, factor loading scores below 0.3 have been censored.

| **Component Matrix^a^** | | | |
| --- | --- | --- | --- |
|  | Component | | |
|  | 1 | 2 | 3 |
| TNF-R1 | .800 |  |  |
| EPHB4 | .750 | -,339 |  |
| IL-18BP | .750 | -.350 |  |
| LTBR | .746 |  |  |
| PLC | .742 |  |  |
| AXL | .733 | -.347 |  |
| U-PAR | .728 |  |  |
| TNF-R2 | .708 | -.309 |  |
| ALCAM | .707 |  | .485 |
| IGFBP-7 | .704 |  | -.386 |
| CD93 | .704 | -.399 |  |
| IL-1RT1 | .696 | -.319 |  |
| TNFSF13B | .687 |  |  |
| TLT-2 | .670 | .463 |  |
| GRN | .670 |  |  |
| SPON1 | .653 |  |  |
| CDH5 | .651 | -.370 |  |
| EGFR | .643 |  |  |
| TFPI | .643 |  |  |
| MCP-1 | .639 |  |  |
| FAS | .637 |  |  |
| MMP-2 | .636 | -.359 |  |
| Notch 3 | .633 | -.353 |  |
| ITGB2 | .625 |  |  |
| KLK6 | .625 |  |  |
| CNTN1 | .625 |  |  |
| CXCL16 | .620 |  |  |
| OPN | .620 |  |  |
| MEPE | .616 |  |  |
| uPA | .599 |  | -.344 |
| Gal-3 | .589 |  | .308 |
| TR-AP | .587 |  |  |
| MPO | .582 |  | -.357 |
| CD163 | .577 |  |  |
| AP-N | .576 |  |  |
| IL-1RT2 | .569 |  |  |
| ICAM-2 | .568 |  |  |
| CTSZ | .553 |  | .316 |
| SHPS-1 | .548 |  |  |
| PRTN3 | .535 |  |  |
| PGLYRP1 | .524 |  |  |
| AZU1 | .515 |  | -.412 |
| IL2-RA | .508 |  |  |
| SELE | .497 |  |  |
| IL-6RA | .497 |  |  |
| CHI3L1 | .493 |  |  |
| COL1A1 | .489 |  | -.392 |
| RETN | .471 |  |  |
| PCSK9 | .464 |  |  |
| PI3 | .446 |  |  |
| MB | .442 |  |  |
| LDL receptor | .436 |  |  |
| TNFRSF10C | .411 |  |  |
| PON3 | .392 |  |  |
| CCL16 | .376 |  |  |
| DLK-1 | .372 |  |  |
| Gal-4 | .355 |  |  |
| TIMP4 | .346 |  |  |
| MMP-9 | .340 |  |  |
| PSP-D |  |  |  |
| CCL24 |  |  |  |
| TR |  |  |  |
| SCGB3A2 |  |  |  |
| Ep-CAM |  |  |  |
| CHIT1 |  |  |  |
| JAM-A |  | .903 |  |
| CASP-3 |  | .902 |  |
| PDGF subunit A |  | .889 |  |
| PECAM-1 |  | .880 |  |
| PAI | .320 | .865 |  |
| SELP |  | .858 |  |
| GP6 | .337 | .850 |  |
| TNFRSF14 | .513 | .792 |  |
| BLM hydrolase | .486 | .761 |  |
| vWF |  | .648 |  |
| t-PA |  | .633 | -.310 |
| CTSD | .565 | .599 |  |
| CSTB | .468 | .593 |  |
| IL-17RA | .447 | .569 |  |
| TFF3 |  |  | .880 |
| GDF-15 | .350 |  | .602 |
| RARRES2 | .498 | .436 | .500 |
| IGFBP-2 | .387 |  | -.499 |
| IGFBP-1 |  |  | .437 |
| OPG | .413 |  | .437 |
| MMP-3 | .341 |  | -.412 |
| ST2 | .396 |  | -.407 |
| CCL15 |  |  | .405 |
| CPA1 |  |  | .330 |
| FABP4 | .303 |  | .310 |
| NT-proBNP |  |  | .308 |
| CPB1 |  |  | .303 |
